# The human placental proteome is affected by maternal smoking

**DOI:** 10.1016/j.reprotox.2016.05.009

**Published:** 2016-08

**Authors:** Pasi Huuskonen, Maria R. Amezaga, Michelle Bellingham, Lucy H. Jones, Markus Storvik, Merja Häkkinen, Leea Keski-Nisula, Seppo Heinonen, Peter J. O’Shaughnessy, Paul A. Fowler, Markku Pasanen

**Affiliations:** a School of Pharmacy, Faculty of Health Sciences, University of Eastern Finland, FIN-70211, Kuopio, Finland; b Division of Applied Medicine, Institute of Medical Sciences, University of Aberdeen, Foresterhill, Aberdeen AB25 2ZD, UK; c Institute of Biodiversity, Animal Health & Comparative Medicine, College of Medical, Veterinary & Life Sciences, University of Glasgow, Glasgow G61 1QH, UK; d Department of Obstetrics and Gynaecology, Kuopio University Hospital, FIN-70211 Kuopio, Finland

**Keywords:** Foetus, Maternal smoking, Metabolism, Placenta, Proteomics, Steroid hormones

## Abstract

Detrimental effects of maternal smoking on the term placental proteome and steroid-metabolizing activities, and maternal hormone levels, were studied by using seven non-smoker and seven smoker placentae. Smoking significantly affected 18% of protein spots. The functional networks affected were i) cell morphology, cellular assembly and organization, cellular compromise (15 hits) and ii) DNA replication, recombination, and repair, energy production, nucleic acid metabolism (6 hits). Smoking significantly up-regulated such proteins as, SERPINA1, EFHD1 and KRT8; and down-regulated SERPINB2, FGA and HBB. Although maternal plasma steroids were not significantly altered, the catalytic activity of CYP1A1 was increased whereas CYP19A1 activity was reduced by smoking. Furthermore, transcript expression of *CYP1A1* and *CYP4B1* were induced while *HSD17B2, NFKB* and *TGFB1* were repressed by smoking. The observed smoking induced wide-spread changes on placental proteome and transcript levels may contribute to the lowered birth weights of the new-born child and placenta.

## Highlights

• The effects of maternal smoking on the term placental proteome was studied.
• Maternal smoking significantly affected 18% of protein spots.
• Maternal smoking affects systems controlling the development and function of placenta.
• The observed placental changes may contribute to the lowered birth weights.

## 1 Introduction

Tobacco smoke contains over >7000 [1] harmful substances that can have direct effects on placental and foetal cell proliferation and differentiation. Many of these substances, such as polycyclic aromatic hydrocarbons, readily pass through the placental barrier into the foetal compartment [2]. Maternal smoking increases the risk of miscarriage, ectopic pregnancy, placenta praevia and foetal orofacial clefts in early pregnancy and foetal growth restriction, placental insufficiency, placental abruption, premature rupture of placental membranes and preterm delivery in late pregnancy [3], [4]. Long-term, prenatal exposure to smoking is a major risk factor for respiratory ailments in children [5], and it may impair their innate and adaptive immunity [6]. In addition, the risks for childhood and adulthood obesity [7], cardiovascular disease [8] and impaired fertility [9] in the offspring are increased by maternal smoking.

Xenobiotic-induced oxidative stress, including maternal smoking, results in placental-associated syndromes and structural changes in placenta, such as increased villous mesenchymal collagen content, trophoblastic membrane thickening, decreased vascularization, villous arteriole oedema and basal plate calcification [3]. Such alterations can result in impaired trophoblast proliferation, differentiation and regulation, as well as disturbed mRNA transcript levels and altered activities of xenobiotic and steroid metabolizing enzymes [10], [11]. This highlights the need for a systematic analysis of placental proteins altered by maternal smoking. In addition, since the term placenta is one of the few tissues that can be used to assess the developmental environment of the newborn child, it would be of considerable value if markers of placental xenotoxicant exposure could be identified.

In this proof of concept study, we selected a 2-D gel approach to enable us to also examine isoform changes, which have functional implications, as well as expression changes in placental proteins following smoke-exposure, as previously published for the human foetal liver [12].

## 2 Materials and methods

### 2.1 Human placental tissue

Fourteen full-term human placentae were obtained, with approval of the local ethic committee, from Kuopio University Hospital, Finland and conducted according to the principles of the Declaration of Helsinki. Maternal smoking was confirmed with a questionnaire during pregnancy and confirmed by measurement of maternal cotinine levels using a liquid chromatography and mass spectrometry (LC–MS) method with a lower sensitivity of 2 ng/ml [13]. Activities associated with the diagnostic placental enzymes 7-ethoxycoumarin *O*-deethylase (ECOD) and 7-ethoxyresorufin *O*-deethylase (EROD) were determined as a measure of smoking-induced cytochrome P450 subfamily 1A1 (CYP1A1) activity. All placentae, except for three non-smoker placentae, were obtained from vaginal deliveries and were collected immediately after delivery. After delivery, connective tissue and coagulated blood were removed from the placenta and placental tissue was rinsed in cold saline solution. Small pieces (5–10 g) including the central area of the placenta were excised, frozen in liquid nitrogen and stored at −80 °C. For 2-D gel proteomics, equal amounts of protein from each individual placenta were pooled according to maternal smoking status and then four replicate gels were electrophoresed for each groups separately. For 1-D gels and Western blot, all 14 samples were electrophoresed individually in separate lanes.

### 2.2 Measurement of placental enzyme activity

EROD activity was measured to determine the induction of CYP1A1 according to Burke et al. [14]. The activity of ECOD was measured as a general marker for placental CYP-associated activities using the method of Greenlee and Poland [15]. Aromatase (AROM) activity was measured to determine CYP19A1 steroid-metabolizing activity by the method of Pasanen [16]. Microsomal UDP-glucuronosyltransferase (UGT) activity was measured using the fluorescent microplate method [17] while glutathione S-transferase (GST) activity was measured by the method of Habig et al. [18].

### 2.3 Quantification of serum steroid hormones

Ten maternal steroid hormones (oestrone, testosterone, androstenedione, androstanedione, dehydroepiandrosterone, dihydrotestosterone, progesterone, pregnenolone, 17-OH-progesterone and 17-OH-pregnenolone) were quantified in whole blood samples collected at delivery using a validated liquid chromatography and tandem mass spectrometry (LC–MS/MS) method [19].

### 2.4 Extraction of total RNA

Total RNA was extracted with a GenElute Kit (Sigma-Aldrich, MO, USA) or with a Qiagen AllPrep mini kit (Qiagen Ltd., Crawley, UK) followed by DNase treatment (Ambion Turbo DNA-Free Kit, Ambion/Life Technologies, USA). The quantity of RNA was analysed with a NanoVue (GE Healthcare, USA) spectrophotometer and the integrity was monitored by gel electrophoresis. The total RNA was stored at −80 °C.

### 2.5 Extraction of protein

Proteins were extracted from placentae with a Qiagen AllPrep DNA/RNA/Protein mini kit (Qiagen Ltd., Crawley, UK), see Ref. [20]. Tissues were homogenized following the manufacturer’s instructions with two main modifications: (i) the addition of protease inhibitors (Protease Inhibitor Cocktail, Sigma-Aldrich Company Ltd., Gillingham, UK) to the lysis buffer (RLT) to maximize protein integrity and yield and (ii) the re-suspension of protein pellets in Modified Reswell Solution [21]. Reproducibility of protein recovery and quality is shown in Supplementary Fig. 1.

### 2.6 Quantitative real-time PCR

cDNA was synthesized with the First-Strand cDNA synthesis technique (M-MuLV Reverse Transcriptase, Fermentas/Thermo Scientific, USA) and 2 μg of total RNA was used in each synthesis. Real-time PCR was performed using Taqman primer probe sets (Applied Biosystems/Life Technologies, USA) or SYBR (Brilliant II, Agilent technologies). Gene expression was normalized with β-actin (ACTB) and each sample were measured in triplicate. The commercial probes and primers used are presented in Supplementary Table 1.

### 2.7 Proteomics

**Fig. 1:**
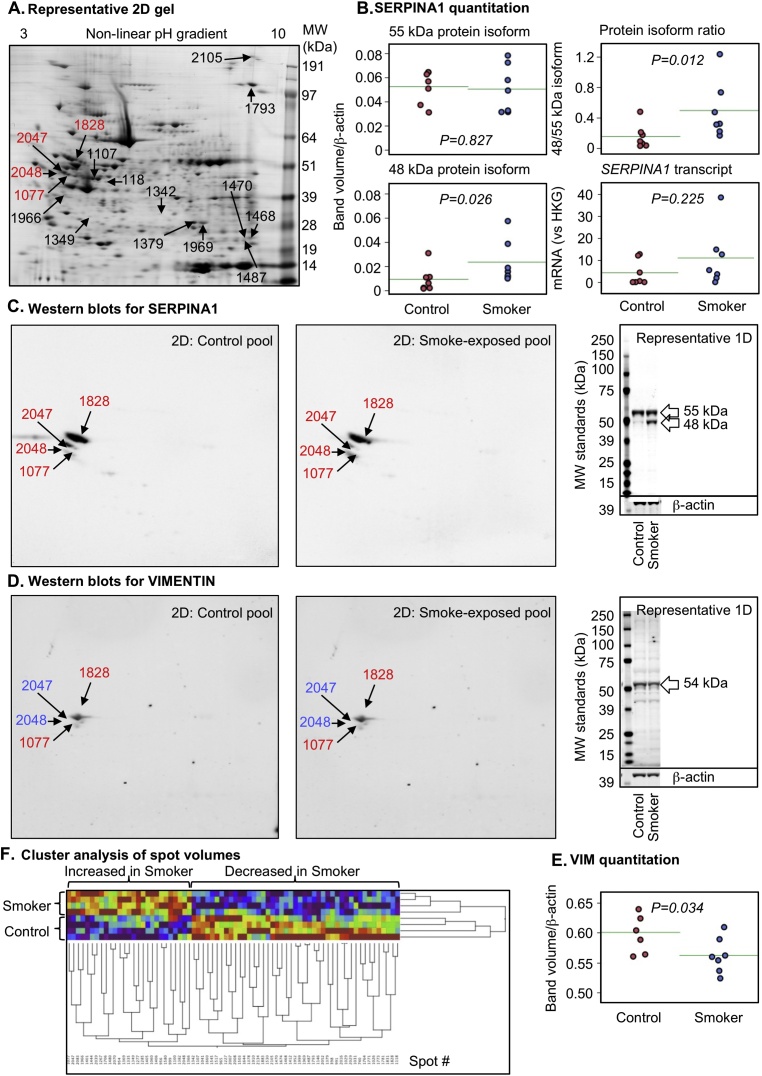
Proteomic analysis of the human placenta at term. (A) Representative 2-D SDS PAGE showing significantly altered protein spots following significant smoke-exposure (*p *< 0.05, ≥1.2-fold change). The spot numbers in red denote the spots shown in (C) and (D). Cleaved SERPINA1 (1-D Western blot of individual placental samples, n = 7/group), but not intact protein or transcript, was significantly increased in smoke-exposed placentae (B). 2-D Western blot (using the same protein pools as used for the proteomic analysis) (C) confirmed that SERPINA1 was present in multiple protein spots. VIM was significantly increased according to proteomic analysis but 2-D Western blot (D) demonstrated that the blue highlighted spots did not overlap with the primary antigen and total immuno-recognised VIM was decreased in smoke-exposed placentae, as shown by 1-D Western blot of individual placental samples (E). (F) Cluster analysis of the significantly altered 2-D protein spot volumes demonstrates separation of the non-smoker and smoke-exposed groups. (For interpretation of the references to colour in this figure legend, the reader is referred to the web version of this article.)

For small-scale or proof of concept studies where proteomics is used, we have found that pooling samples to build-in biological variation, with quadruplicate gel electrophoresis to contribute the technical variation is a successful design. Therefore, protein pools, to which individual placentae contributed equally in term of protein quantity, were prepared for each experimental group and soluble proteins separated by 2-DE in quadruplicate using commercial 7 cm gels, as previously described [21]. The gels were stained with Colloidal Coomassie Brilliant Blue, scanned and analysed with Progenesis SameSpots software, V6.01 (Nonlinear Dynamics, Newcastle, UK). The software was used to combine the gel quadruplicates and to calculate fold-changes and significance. Briefly, a single reference gel for the whole experiment was selected from one of the four control gels (Fig. 1A), based on image quality, this also served as the reference gel for the control group while the highest quality gel in the smoking group served as the reference gel for that group. Spots around the edge of the gel were excluded and spots were detected based on all detected spots in all 8 gels followed by automatic alignment, first to reference gels for each group separately and then to the experiment reference gel. The alignments were checked manually and poorly matched spots within groups were excluded from the analysis. Additional manual editing was not required. Spots on all gels were normalised (no gels were excluded from the analysis) using the automatic normalisation routine of the software (see http://totallab.com/samespots-technical-details/for further information). Spots with fold-differences of ≥1.2-fold at p < 0.05 were included in the overall analysis. Differentially-expressed protein spots were selected for spot cutting once statistical significance (provided within SameSpots by ANOVA of log-normalised spot volumes) had been independently verified by the authors (see Statistical Analysis below) and all were consistently expressed across all 8 gels with fold-differences of ≥1.5-fold at p < 0.05. Molecular masses and pI values of spots of interest were estimated from separate gels electrophoresed with pH and MW markers. Proteins in the gel pieces were digested with trypsin and analysed see Ref. [21] for details. Mass lists in the form of Mascot Generic Files were created automatically and used as the input for Mascot MS/MS Ions searches of the NCBInr database using the Matrix Science web server (www.matrixscience.com). The default search parameters used were: Enzyme = Trypsin, Max. Missed cleavages = 1; Fixed modifications = Carbamidomethyl (C); Variable modifications = Oxidation (M); Peptide tolerance ± 1.5 Da; MS/MS tolerance ± 0.5 Da; Peptide charge = 2+ and 3+; Instrument = ESI-TRAP. Statistically significant MOWSE scores and good sequence coverage were considered to be positive identifications.

### 2.8 1-D gel electrophoresis and 1-D and 2-D western blot

Individual (n = 7/group) placental protein extracts were electrophoresed (30 μg protein/lane) on 26-lane 1-DE 4–12% Bis-Tris gels (Invitrogen Ltd, Paisley, UK) under reducing conditions (MOPS buffer, Invitrogen) [20], [21]. The same pooled placental protein samples as used for proteomics (section 2.7 above) were also used to generate additional 2-D gels. Both 1-D and 2-D gels were transferred to Immobilon™-FL membranes (Millipore (UK) Ltd, Watford, UK) as described previously [20], [21]. Odyssey Two-Colour Protein Molecular Weight Markers (LI-COR Biosciences UK Ltd, Cambridge, UK) were electrophoresed in 3 lanes of every gel. The membranes were blocked (1 h, room temperature) with (Odyssey Blocking Buffer, 927–4000: LICOR +PBS) and were incubated with primary antibodies (diluted in blocking buffer + PBST) at 4 °C overnight: (i) Transgelin (TAGLN2) 0.001 μg/ml, goat, AHP2034, ABD Serotec, Kidlington, Oxfordshire, UK; (ii) Carbonic anhydrase-1 (CA1) 1:1000, mouse, H00000759-A01, Abnova, Stratech Scientific Ltd, Newmarket, Suffolk, UK; (iii) Peroxiredoxin-1 (PRDX1) 1:500, rabbit, PA0007, LabFrontier, no longer available; (iv) Alpha-1-antitrypsin (SERPINA1) 1:500, rabbit, HPA00129, Sigma-Aldrich Company Ltd, Gillingham, Dorset, UK. Membranes were also immuno-stained with an anti-ACTB (anti β-actin) load control from a different species (1:5000, mouse, ab8226, Abcam Plc, Cambridge, Cambridgeshire, UK or 1:10000, rabbit A5060, Sigma-Aldrich). IRDye^®^ infrared secondary antibodies were supplied by LI-COR. Protein bands were visualized using the LI-COR Odyssey^®^ Infrared Imaging System and the resulting electronic images were analysed using TotalLab TL120 software (v2008.1; Nonlinear Dynamics Ltd, Newcastle-upon-Tyne, UK) to determine the molecular weights and band volumes. The band volumes of ACTB were compared between groups to check the validity of this load control for placentae from the non-smoking and smoking groups. 1-D load control SDS PAGE gel of all placental protein extracts studied is presented in Supplementary Fig. 1.

### 2.9 Statistical and pathway analysis

Statistical significance of demographic, biochemical and hormonal data was carried out by Mann-Whitney *U*-test and data are presented as mean ± SD, and *P*-values <0.05 were considered statistically significant. The normality of proteomic data distribution was tested with the Shapiro-Wilk test and where distribution of data was skewed, they were log-transformed prior to analysis or analysed using the non-parametric Wilcoxon test. The 1-DE Western blot band volumes (normalized relative to ACTB expression separate for each lane) and normalized spot volumes (% of total spot volume for each gel separately) were compared in control and smoke-exposed groups, using one-way ANOVA. Statistically significant differences in log-transformed, normalized volumes were an important determinant of the spots selected for identification by LC/MS-MS. Unless otherwise stated, data are presented as mean ± SEM. Where fold-changes are presented, a positive value indicates an increase, and a negative value is a reduction, relative to controls. Proteins and transcripts exhibiting treatment-specific alterations in expression were analysed using Ingenuity Pathway Analysis (IPA, Ingenuity Systems, http://www.ingenuity.com), as described previously [12], [21], [22].

## 3 Results

### 3.1 The weights of newborns were reduced by maternal smoking

The mean birth weight of neonates was significantly (p < 0.01) reduced in the smoking group compared with those in the non-smoking group (Table 1). In the smoking group, a non-significant trend (p = 0.165) for reduced placental weight was observed. Women were significantly younger (p < 0.05) in the smoking group compared to the non-smoking group (Table 1).

### 3.2 Maternal smoking alters placental metabolizing enzyme activity

In the smoking group, placental enzyme activities ECOD and EROD were significantly induced (p < 0.01) and AROM levels significantly repressed (p < 0.05) compared to the non-smoking group (Table 2). However, GST and UGT were not altered. A significant increase was observed in placental mRNA transcript levels of *CYP1A1* and *CYP4B1* (Table 3) in the smoking group (p < 0.01 and p < 0.05, respectively).

### 3.3 Smoking has little effect on maternal plasma steroid hormone levels

Maternal plasma steroid hormone concentrations demonstrated a wide inter-individual variation without any significant alterations between the smoking and non-smoking groups (Table 4). In the smoking group one mother displayed, low estrogen and progestogen levels, and exceptionally high androgen levels although her placental AROM activity was similar to the others. Of the 10 steroidogenic enzyme transcripts quantified (Table 3), only hydroxysteroid dehydrogenase 17B2 (*HSD17B2)* displayed a significantly decreased expression in the smoking group (p < 0.05).

### 3.4 The placental proteome is disturbed by maternal smoking

**Fig. 2:**
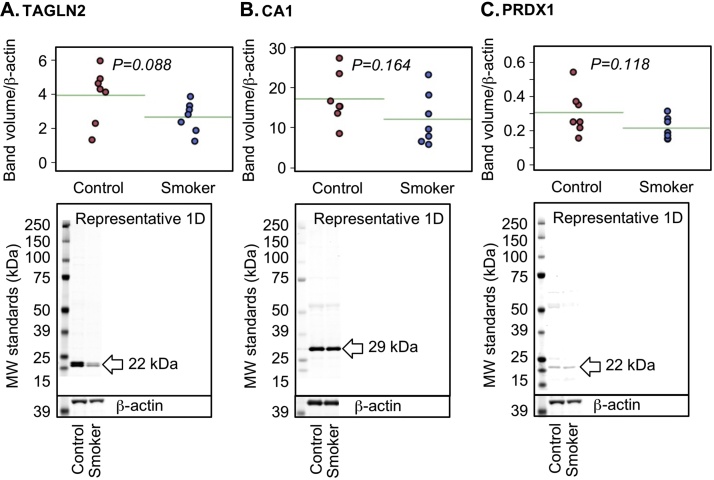
1-D Western blot of individual placental samples (n = 7/group) showed a similar trend as found in the proteomic findings for (A) TAGLN2, (B) CA1 and (C) PRDX1, but this was not statistically significant.

**Fig. 3:**
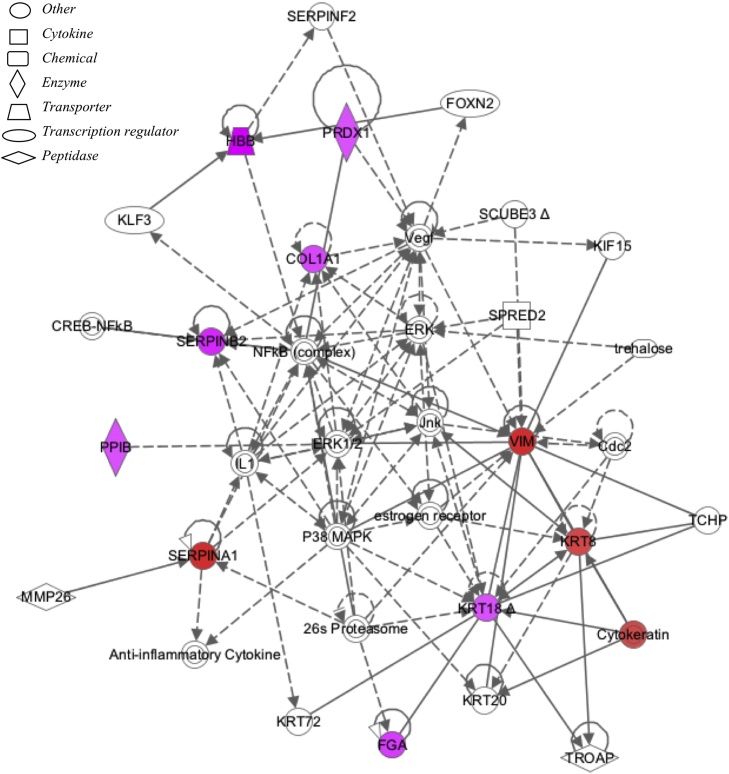
Network 1 (Cell Morphology, Cellular Assembly and Organization, Cellular Compromise) identified from maternal smoking effects on the proteome of the term human placenta. Networks were generated through the use of Ingenuity Pathway analysis. The intensity of color represents degree of up-regulation (purple) and down-regulation (red). A key to the identity of the node shapes is included in the figure. Dashed and solid lines represent indirect and direct interactions respectively. Lines without arrows indicate binding while closed arrows indicate action of first on second node and open arrows indicate translocation from first to second node. (For interpretation of the references to colour in this figure legend, the reader is referred to the web version of this article.)

**Fig. 4:**
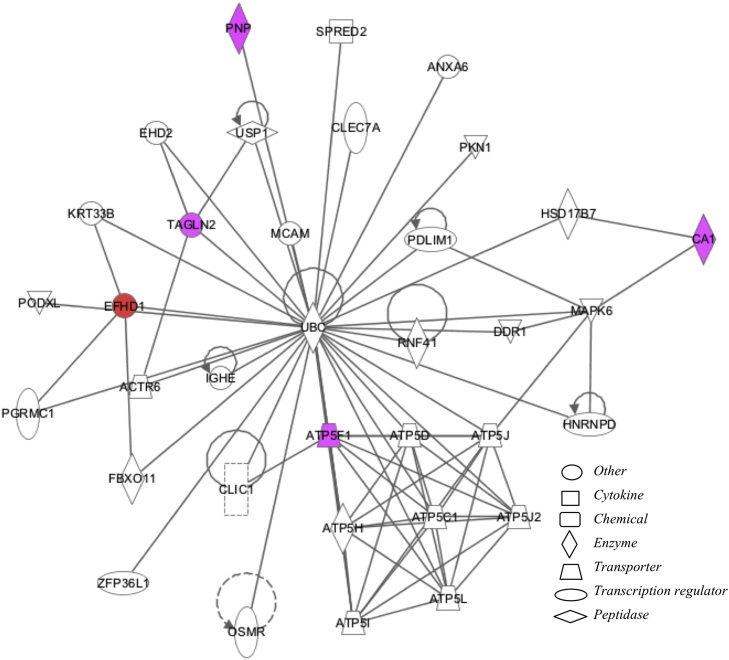
Network 2 (DNA Replication, Recombination, and Repair, Energy Production, Nucleic Acid Metabolism) identified from maternal smoking effects on the proteome of the term human placenta. Networks were generated through the use of Ingenuity Pathway analysis. The intensity of color represents degree of up-regulation (purple) and down-regulation (red). A key to the identity of the node shapes is included in the figure. Dashed and solid lines represent indirect and direct interactions respectively. Lines without arrows indicate binding while closed arrows indicate action of first on second node and open arrows indicate translocation from first to second node. (For interpretation of the references to colour in this figure legend, the reader is referred to the web version of this article.)

Maternal smoking significantly affected 72 protein spots (p < 0.05, ≥1.2-fold change) out of 392 protein spots included in the study (based on quality criteria of size and reproducibility). Maternal smoking increased 27 and decreased 45 protein spot volumes (Fig. 1A). Sixteen of the most altered and consistent of these protein spots were identified by using LC–MS/MS (Table 5). Because protein spots can contain more than one protein and different protein isoforms may have different migration patterns [22], selected proteins with likely roles in the placenta were further analysed by Western blot. Maternal smoking significantly increased the cleaved (48 kDA) form of α-1-antitrypsin (SERPINA1) but not the 55 kDa full-length protein or transcript (Fig. 1B, C). Vimentin (VIM) was identified in two protein spots, which had significantly increased spot volumes in the smoking group (Table 5 and Fig. 1D). In the 2-D Western blot of VIM the antibody used marginally overlapped with the spots in blue in Fig. 1D. Therefore, VIM is a minor component of spot # 2047 and 2048 (Fig. 1D, peptide intensity ration 2.30:1.00 in favour of SERPINA1) and, in contrast, immunodetected VIM protein was significantly decreased (Fig. 1E). A non-significant trend for decreasing immune-detectable protein expression of carbonic anhydrase 1 (CA1), peroxiredoxin 1 (PRDX1) and transgelin 2 (TAGLN2) was observed among smokers (Fig. 2), similar to the proteomics findings (Table 5). By using Ingenuity Pathway Analysis (IPA), the proteomic findings shown in Table 5 were mapped to two networks: I) Cell Morphology, Cellular Assembly and Organization, Cellular Compromise (Fig. 3, Supplementary Table 2) and II) DNA Replication, Recombination, and Repair, Energy Production, Nucleic Acid Metabolism (Fig. 4, Supplementary Table 2). These pathways mapped to toxicological and biological functions shown in Supplementary Table 3 and included liver function and disease and cell death, survival and function. Canonical pathways in the term human placental proteome affected by maternal smoking (Supplementary Fig. 2) include several detoxification and metabolic pathways as well as signalling and blood function pathways. Based on the pathway analysis, eight mRNA transcripts associated with placental consequences of smoking, intra-uterine growth restriction, or with SERPINA1, were quantified by qPCR. Among the transcripts measured (Table 3 “other pathways”), only nuclear factor kappa-light-chain-enhancer of activated beta cells (*NFKB)* and transforming growth factor, beta 1 (*TGFB1*) were statistically significantly decreased.

## 4 Discussion

In this study, placental proteins relating to two main functional protein networks (i: Cell Morphology, Cellular Assembly and Organization, Cellular Compromise; ii: DNA Replication, Recombination, and Repair, Energy Production, Nucleic Acid Metabolism) were significantly affected by maternal smoking in the human term placentae. The protein affected included constructs of haemoglobin subunits and protective proteases (e.g. SERPINA1) and proteins directly involved in cellular structure or carbon dioxide metabolism.

SERPINA1 is a protease inhibitor that belongs to the serine proteinase (serpin) superfamily and protects against inflammatory cells initiated cell damage [24]. In addition, there are multiple forms of SERPINA1, which are associated with the expression of the different pro-inflammatory molecules [25]. In the present study, the expression of the cleaved 48 kDa form of SERPINA1 (NP_000286) protein was significantly lower in the smoking group compared to non-smoking group (fold-change −1.5). However, maternal smoking significantly increased (fold-change +1.6 to +1.8) expression levels of three other SERPINA1 protein variants (1QMB_A, AAH15642 and AAB59495). This suggests that maternal smoking can disturb transcript splicing in placenta. According to the literature there are contradictory data based on placental SERPINA1 protein expression levels. This is due to the differences in risk factors for pregnancy: SERPINA1 was increased in placentas of obese mothers [26] and decreased in preeclamptic mothers [27]. SERPINA1 is increased in the lung and sputum of smokers [28], which, together with our data and known effects of smoking on the placenta [29], suggests that specific SERPINA1 isoforms could serve as a biomarker for oxidative stress. It is also possible that the alterations in placental SERPINA1 expression levels reflect responses to external stress factors and, therefore, could reflect the overall maturation process or well-being of the foeto-placental unit. Strikingly, our data showed good agreement with a recently published study on the effects of growth restriction on the human placenta in which SERPINA1 was increased and SERPINB2 decreased [30]. The identification of multiple proteins in other spots (e.g. #1468) and their isoforms (e.g. VIM) will require follow-up in further studies because of the potential functional importance of these differences [23].

Other placental proteins affected by maternal smoking included carbonic anhydrase 1 (CA1), haemoglobin subunit β (HBB) and plasminogen activator inhibitor 2 (SERPINB2). The expression of SERPINB2 protein was significantly decreased in the placentae of smokers. Biologically SERPINB2 is a coagulation factor produced in the human placenta. It inactivates urokinase plasminogen activator (uPA) and tissue plasminogen activator (tPA) [31]. In line with our data, SERPINB2 transcript was significantly down-regulated in a study performed with umbilical cords of maternal smokers [32]. Potentially, the down-regulation of SERPINB2 will lead to increased risk for haemorrhage during pregnancy. As a consequence, this may increase risk for gestational complications such as placental abruption in smokers.

HBB expression was reduced significantly in the placentae from smoking women. Lower levels of proteins associated with haemoglobin synthesis could lead to inadequate oxygen transfer to the foetus and increase risk of intrauterine oxygen depletion and foetal growth retardation seen in maternal smoking. Similarly, placentae from severe preeclamptic mothers have demonstrated comparable decrease in haemoglobin–β-chain levels [27]. In contrast, HBB, HBA and HBG levels were significantly elevated in proteomic studies of: (i) cord blood from maternal smokers [33] and (ii) the effects of growth restriction on the human placenta [30]. Carbonic anhydrases are key components in transporting carbon dioxide from tissues to circulation, and maintaining the acid-base balance in tissues. Therefore, the significant reduction in placental levels of CA1 combined with the repressed synthesis of haemoglobin subunits in the smoking group would be expected to exert synergistic effects leading to impaired oxygen supply and acid-base imbalance.

Levels of transcripts identified by IPA analysis of the smoking-responsive placental proteome, specifically those encoding *NFKB* and *TGFB1*, were significantly decreased in the placenta by maternal smoking. Oxidative stress is usually associated with increased NFKB, which also mediates responses to hypoxia and up-regulates cellular responses to inflammation in placental cells [34]. Failure of NFKB levels to increase in placentae from maternal smokers may limit the ability of the placenta to protect itself from the outcomes rising from the oxidative stress [35]. However, this will require further study due to the occurrence of at least two NFKB isoforms. TGFB1 is a multifunctional cytokine that regulates placental trophoblast cell proliferation and differentiation, and hormone production [36]. A decrease in transcript levels has, therefore, the potential to affect several aspects of placental function and growth and to increase the risk of placental insufficiency.

Maternal smoking also affected several intra-cellular structure-related members of the placental proteome. Significant decreases in the levels of collagen (COL1A1), fibrinogen (FBA), keratin (KRT18), and VIM (the latter measured by Western blot) occurred if the mother smoked. The expression changes in these specific placental structural proteins may reflect growth restriction, structural and functional consequences, e.g. placental abruption, insufficiency, premature rupture of placental membranes and preterm delivery [3]. VIM is a component of the cytoskeleton, which is required to maintain cellular integrity and its expression levels were altered in women suffering preterm labour [37]. However, in the current study no difference was seen in terms of pregnancy duration between smoking and non-smoking mothers.

Maternal smoking altered several functional enzyme activities in the placental samples (Table 3). The placental monooxygenase activities, EROD and ECOD, were both induced among smokers’ placentae probably by constituents of the cigarette smoke [10]. CYP19A1 activity was also decreased in the smokers’ placentae [38], which could lead to reduced placental oestrogen synthesis capacity and housekeeping functions. Maternal smoking also led to an induction of *CYP1A1* and *CYP4B1* mRNA levels and a repression of *CYP19A1* mRNA levels. These findings agree with our previous study [10], although gross CYP1A1 and CYP19A1 protein levels were not significantly altered in our Western blot analysis (data not shown). Among the hydroxysteroid dehydrogenases, the mRNA of *HSD17B2* was repressed in the smoker group. HSD17B2 converts testosterone to androstenedione, estradiol to estrone, and 20-alpha-dihydroprogesterone to progesterone. In human placenta *HSD17B2* is located in the endothelial cells lining the foetal compartment. It has been hypothesized that HSD17B2 acts as a barrier to reduce the rate of estradiol secretion into the foetal circulation [39] and it is possible, therefore, that maternal smoking alters circulating foetal estradiol levels. This is supported by our recent publication demonstrating significantly increased second trimester estrogen levels in human foetal plasma [40]. The maternal serum steroid hormone levels displayed wide inter-individual variations in both of the studied groups. Despite the changes observed in the placental hormone metabolism no significant alterations were detected in maternal serum steroid hormone levels, although cigarette smoking has been demonstrated to affect maternal steroid hormone levels and reduce estriol levels in the cord blood [41].

All pregnancies included in the present study were classified as clinically “normal” and resulted in healthy babies and none of the women underwent long-term medication during gestation. When the smoking and non-smoking groups were analysed, foetal sex was added as a covariate and there were no significant interactions between foetal sex and maternal smoking for any of the reported data. Sample size in this study was small but was sufficient to confirm the epidemiological findings that maternal smoking was associated with lower birth weights [4]. One limitation of the study is that the women were significantly younger in the smoking group compared to non-smoking group. In general, smoking mothers are younger than non-smokers as demonstrated in the USA where 20% of pregnant women aged <25 years smoke and 9% of those aged >35 years [42]. Our data agrees with the previous findings that the mean placental weight was reduced in the smoker group [43]. However, also maternal age may influence placental weight or morphometry [44]. The changes observed in the present proteomic analysis will not directly reflect to the weight or size or histology of placenta as reported in other studies [45]. Nonetheless our data support the working hypothesis that maternal smoking affects multiple fundamental cellular systems controlling the growth, development and function of placenta. This is emphasized in Supplementary Table 3 where the biological functions with the largest number of members with overlapping proteins identified in the present study were found in the categories of cell growth, survival, movement and death. One caveat of course is that by looking at the term placenta we cannot determine when over the 9 months of pregnancy, the changes in placental function occurred. This does not reduce the importance of studying the term placenta both due to its own intrinsic biomedical importance and because it is a readily accessible product of conception that can act as a cumulative read-out for the whole of gestation.

## 5 Conclusions

In conclusion, based on the literature, altogether 4239 proteins have been identified in human placental tissue, covering around 21% of the human proteome [46]. In the present study we show that maternal smoking significantly affected term placental levels of at least 70 proteins. The two functional protein networks affected were: (1) cell morphology, cellular assembly and organization; (2) DNA replication, recombination, and repair, energy production, nucleic acid metabolism. The clinical significance of increased SERPINA1 expression among smokers’ placentae is not known but its known functions suggest significant deviation in placental function in cigarette smoking women. The changes described at the placental proteome and transcript levels may reflect the increased oxidative stress due to maternal smoking, resulting in lowered birth weight of a new born child and placenta. Based on the concept, developmental origin of diseases, it remains unresolved whether the maternal smoking-induced placental proteomic changes reflect to the wellbeing or morbidity of children later in life.

## Conflict of interest

The authors declare that they have no conflict of interest.

## Transparency document

Transparency Document

## Figures and Tables

**Table 1:** Demographic and perinatal data of the non-smoking and smoking groups.

Variable	Non-smokers^a^	Smokers^a^	*p*-Value^b^
Maternal age (years)	32.14 ± 6.28	26.00 ± 3.74	0.026*
Number of previous pregnancies	2.43 ± 0.53	2.14 ± 1.07	0.456
Duration of pregnancy (weeks)	39.65 ± 0.66	39.21 ± 1.60	1.000
Birth weight (g)	3719.29 ± 196.86	3202.50 ± 309.38	0.002**
Placental weight (g)	680.83 ± 63.12	588.57 ± 73.35	0.165

a Non-smokers = 4 male, 3 female; Smokers = 7 males.

b As determined by Mann-Whitney *U* test, Values are shown as mean ± SD. Non-smokers *vs.* smokers.

* *p* < 0.05.

** *p* < 0.01.

**Table 2:** Comparison of the biochemical data between the non-smoking and smoking groups.

Variable	Non-smokers (*n* = 7)	Smokers (*n* = 7)	*p*-value^a^
Blood cotinine (ng/ml)	<2.00^b^	51.51 ± 26.94 (16.78–96.84)	–
EROD (pmol*mg/min)	0.30 ± 0.34 (0.02–0.78)	151.28 ± 180.56 (1.77–516.28)	0.001**
ECOD (pmol*mg/min)	5.50 ± 4.14 (1.93–13.86)	297.38 ± 264.79 (13.02–782.60)	0.001**
AROM (pmol*mg/min)	33.01 ± 7.28 (22.05–45.28)	27.78 ± 31.36 (10.84–98.26)	0.026*
GST (nmol*mg/min)	178.89 ± 36.45 (126.19–219.25)	230.41 ± 85.89 (183.98–418.27)	0.209
UGT (pmol*mg/min)	5.00 ± 1.38 (3.66–7.28)	5.37 ± 1.89 (3.65–8.69)	0.902

Values are shown as mean ± SD and range in brackets below.

EROD = 7-ethoxyresorufin *O*-deethylase, ECOD = 7-ethoxycoumarin *O*-deethylase, AROM = aromatase, GST = glutathione S-transferase, UGT = uridine 5′-diphospho-glucuronosyltransferase.

a As determined by Mann-Whitney *U* test.

b Below limit of quantitation. Non-smokers *vs.* smokers.

* *p* < 0.05.

** *p* < 0.01.

**Table 3:** Relative *ACTB*-normalized expressions of transcripts of interest in the term human placenta. Bold text denotes transcripts where maternal smoking had a statistically significant effect on transcript expression.

Gene	Non-smokers	Smokers	*p*-value^a^
Xenosensors			
*AHR*	1 ± 0.29	1.98 ± 0.42	0.701

Endocrine receptors			
*KISSR*	1 ± 0.44	0.89 ± 0.42	1.000

Steroidogenic enzymes			
*AKR1C3*	1 ± 0.49	1.44 ± 0.81	0.523
*CYP11A1*	1 ± 0.49	0.49 ± 0.45	0.702
*CYP19A1*	1 ± 0.54	0.66 ± 0.71	0.259
*HSD3B1*	1 ± 0.50	0.90 ± 0.60	0.710
*HSD3B2*	1 ± 0.43	1.13 ± 0.47	0.898
*HSD11B2*	1 ± 0.37	0.54 ± 0.65	0.097
*HSD17B1*	1 ± 0.66	0.29 ± 0.38	0.702
***HSD17B2***	**1** **±** **0.32**	**0.15** **±** **0.37**	**0.048**
*HSD17B6*	1 ± 0.77	1.44 ± 0.81	0.705
*POR*	1 ± 0.47	0.43 ± 0.60	0.710

Phase I enzymes			
***CYP1A1***	**1** **±** **0.64**	**142.86** **±** **0.95**	**0.001**
*CYP2B6*	1 ± 0.76	0.26 ± 0.58	0.898
***CYP4B1***	**1** **±** **0.63**	**6.88** **±** **1.45**	**0.026**
*CBR1*	1 ± 0.48	0.83 ± 0.54	0.535
*EPHX1*	1 ± 0.54	3.37 ± 0.49	0.201

Phase II enzymes			
*GSTA1*	1 ± 0.59	1.69 ± 0.67	0.701
*GSTP1*	1 ± 0.28	3.21 ± 0.48	0.159
*GSTT1*	1 ± 0.54	0.60 ± 0.53	0.798
*NQO1*	1 ± 0.95	5.73 ± 0.71	0.201

Other pathways			
*GGT1*	1 ± 0.96	0.01 ± 0.32	0.203
*SERPINA1*	1 ± 0.52	2.59 ± 0.46	0.250
***TGFB1***	**1** **±** **0.28**	**0.28** **±** **0.40**	**0.046**
*FOS*	1 ± 0.40	0.58 ± 0.25	0.999
***NFKB***	**1** **±** **0.42**	**0.22** **±** **0.27**	**0.047**
*WISP2*	1 ± 0.33	1.10 ± 0.35	0.818
*CEBPB*	1 ± 0.50	0.36 ± 0.34	0.798
*IGF1*	1 ± 0.70	0.10 ± 0.56	0.250
*IGF2*	1 ± 0.30	1.17 ± 0.25	0.688

Values are transformed to relative expression ± SEM. Percentual expressions were calculated by comparing smokers against non-smoker expressions, assuming that non-smoker expressions are 1.

a As determined by ANOVA.

**Table 4:** Maternal hormone concentrations for the non-smoking and smoking groups.

Hormone	Non-smokers (*n* = 7)	Smokers (*n* = 7)	*p*-value^a^
Estrone	119.98 ± 56.90 (44.36–216.10)	135.44 ± 88.45 (21.96–248.41)	0.710
Testosterone	0.23 ± 0.13 (0.076–0.385)	0.65 ± 1.07 (0.082–3.042)	0.535
Androstenedione	3.18 ± 0.59 (2.23–3.87)	4.76 ± 4.92 (1.16–15.68)	0.805
Androstanedione	<1.33^b^	<1.33^b^	–
Dehydroepiandrosterone	8.16 ± 3.13 (5.15–13.40)	12.66 ± 5.63 (5.15–21.28)	0.165
Dihydrotestosterone	<0.325^b^	<0.325^b^	–
Progesterone	3189.35 ± 1795.52 (1036.93–5740.57)	3005.66 ± 1179.88 (493.65–4305.20)	1.000
Pregnenolone	97.49 ± 27.46 (50.66–127.96)	109.74 ± 45.92 (17.99–167.78)	0.535
17α-Hydroxyprogesterone	81.66 ± 56.06 (35.83–184.28)	79.74 ± 45.13 (29.61–164.53)	1.000
17α-Hydroxypregnenolone	12.19 ± 4.44 (5.24–19.45)	18.62 ± 10.73 (5.45–32.00)	0.209

Values are shown as mean ± SD and range in brackets below. All units of variables are nM.

a As determined by Mann-Whitney *U* test.

b Below limit of quantitation.

**Table 5:** Identification of proteins in 16 2-D gel spots significantly affected by maternal smoking in human term placenta. The proteins are grouped into two networks according to the Ingenuity Pathway Analysis (IPA) (see Supplementary Table 2).

Spot #	*Gene symbol*	Protein name	Accession #	Fold-change	*p*-value^a^	MW	pI	MOWSE Score
Network 1: Cell Morphology, Cellular Assembly and Organization, Cellular Compromise								
118	*SERPINB2*	Plasminogen activator inhibitor 2	NP_002566	−1.9	0.041	46.8	5.46	528
1828	*SERPINA1*	Alpha-1-antitrypsin	NP_000286	−1.5	0.035	46.9	5.37	974
2047^b^	*SERPINA1*	Alpha-1-antitrypsin (or S variant)	1QMB_A	+1.8	0.004	36.6	5.44	1086
1077	*SERPINA1*	Alpha-1-antitrypsin	AAH15642	+1.8	<0.001	39.1	5.27	942
2048^b^	*SERPINA1*	Alpha-1-antitrypsin	AAB59495	+1.6	0.016	46.8	5.43	716
1468^b^	*PPIB*	Peptidyl-prolyl *cis*-trans isomerase B	AAA52150	−1.5	0.008	22.8	9.33	309
1487^b^	*PRDX1*	Peroxiredoxin 1	NP_002565	−1.5	0.036	22.3	8.27	480
1468^b^	*FGA*	Fibrinogen alpha chain	0501249A	−1.5	0.008	26.6	9.08	444
1470	*FGA*	Fibrinogen alpha chain	0501249A	−1.7	0.002	26.6	9.08	425
2047^b^	*VIM*	Vimentin	AAA61279	+1.8	0.004	53.7	5.03	426
2048^b^	*VIM*	Vimentin	AAA61279	+1.6	0.016	53.7	5.03	724
1793	*COL1A1*	Collagen alpha 1(I) Chain	AAH36531	−1.5	0.005	139.9	5.70	187
2105^d^	*COL1A1*	Collagen alpha 1(I) Chain	AAH36531	−1.6	<0.001	140.0	5.70	125
1966	*KRT8*	KRT8 protein	AAH11373	+1.5	0.017	41.1	4.94	582
1107	*KRT18*	KRT18 protein, cytoskeletal	CAA31377	−1.5	<0.001	47.3	5.27	1337

Network 2: DNA Replication, Recombination, and Repair, Energy Production, Nucleic Acid Metabolism								
1342	*PNP*	Purine nucleoside phosphorylase	NP_000261	−1.6	<0.001	32.3	6.45	684
1379	*CA1*	Carbonic anhydrase 1	NP_001729	−1.5	0.004	28.9	6.59	617
1468^b^	*ATP5F1*	ATP synthase subunit b, mitochondoral	AAH05960	−1.5	0.008	28.9	9.45	218
1487^b^	*TAGLN2*	Transgelin-2	NP_003555	−1.5	0.036	22.5	8.41	450
1969^c^	*HBB*	Hemoglobin subunit beta	2DXM_D	−2.6	0.032	15.9	6.55	643
1349	*EFHD1*	EF-hand domain-containing protein D1	NP_079479	+1.6	0.004	27.0	5.34	408

a As determined using log-normalized spot volumes.

b More than one protein highly likely to be in the spot.

c Poor gel match with MW and pI but sequence coverage of 94% and an ion score of 21 suggests an accurate identification.

d Poor sequence coverage and gel MW match.
