# Supplementary material for: The human placental proteome is affected by maternal smoking
Source: Reprod Toxicol. 2016 Aug;63:22–31. doi: 10.1016/j.reprotox.2016.05.009 (PMC4991937; doi:10.1016/j.reprotox.2016.05.009)
Supplement: Supplementary file 2 [file mmc1.docx]

**The human placental proteome is affected by maternal smoking**

Pasi Huuskonen^a^, Maria R. Amezaga^b^, Michelle Bellingham^c^, Lucy H. Jones^b^, Markus Storvik^a^, Merja Häkkinen^a^, Leea Keski-Nisula^d^, Seppo Heinonen^d^, Peter J. O’Shaughnessy^c^, Paul A. Fowler^b^,

Markku Pasanen^a,*^

^a^ School of Pharmacy, Faculty of Health Sciences, University of Eastern Finland, FIN-70211, Kuopio, Finland

^b^ Division of Applied Medicine, Institute of Medical Sciences, University of Aberdeen, Foresterhill, Aberdeen AB25 2ZD, UK

^c^ Institute of Biodiversity, Animal Health & Comparative Medicine, College of Medical, Veterinary & Life Sciences, University of Glasgow, Glasgow G61 1QH, UK

^d^ Department of Obstetrics and Gynaecology, Kuopio University Hospital, FIN-70211 Kuopio, Finland

*** Corresponding author**

Markku Pasanen, School of Pharmacy, Faculty of Health Sciences, University of Eastern Finland, P.O. Box 1627, FIN-70211 Kuopio, Finland. Phone: +358 40 7199 346. Fax: +358 17 162 424. E-mail: markku.pasanen@uef.fi

**
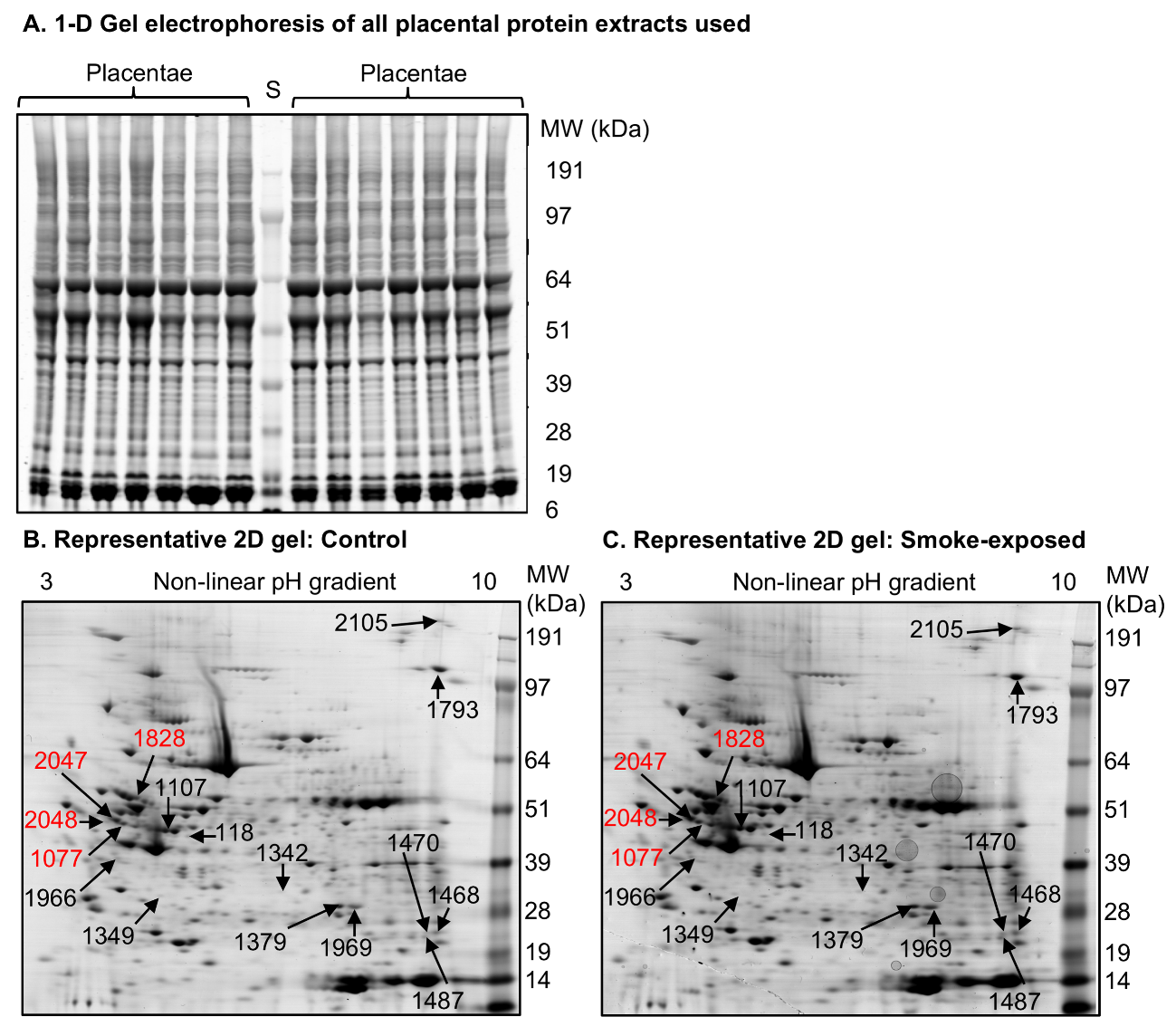
**

**Supplementary Figure** **1.** Representatives 1-D and 2-D electrophoretic gel images. (A) 1-D Coumassie blue stained gel images showing migration patterns for all 14 samples. (B, C) 2-D Coumassie blue stained gel images from pooled samples for (B) Control and (C) Smoke-exposed groups.

**Supplementary Table 1.** Commercial probes (Applied Biosystems/Life Technologies, USA) and the forward (F) and reverse (R) primers for RT-qPCR.

| **Gene** | **Product code / Primers** |  |
| --- | --- | --- |
| *ACTB* | 4326315E |  |
| *CYP1A1* | Hs00153120_m1 |  |
| *CYP4B1* | Hs00167970_m1 |  |
| *CYP19A1* | Hs00240671_m1 |  |
| *HSD3B1* | Hs00426435_m1 |  |
| *HSD11B2* | Hs00388669_m1 |  |
| *HSD17B1* | Hs00166219_g1 |  |
| *CBR1* | Hs00156323_m1 |  |
| *AHR* | aatacagagttggaccgtttggctagc  tggcctccgtttctttcagtaggg |  |
| *AKR1C3*  *CYP11A1* | aacgtgcaggtttttgagttccagttg  ggtagacatcaggcaaagccctcc  tttttgcccctgttggatgca  ccctggcgctccccaaaaat |  |
| *CYP2B6*  *HSD3B* | cagcgccaccctaacacccat  ggcctcggtcccaggtgtacc  ccacaccgcctgtatcattgatgtct  taggagttgggcccggctacct |  |
| *HSD17B1* | ggacgcgtgttggtgaccg  actgggccgcactcgatcag |  |
| *HSD17B2* | atggaaaggctggcatcttatggct  cagcttttcccacttgtcactggtg |  |
| *HSD17B6*  *POR* | tctgacaggctggagacggtgac  gtgttcagccactcacataaggtaattgg  ttttcagcatgacggacatgattctgt  tttcttcatcttttccacaaagctgctc |  |
| *EPHX1* | tggcggaatgaatttgactggaa  cagccgtgcaccatcagcaag |  |
| *GSTA1* | gcaacaagctgagccgggct  tgggctgccaggctgtagaaac |  |
| *GSTP1*  *GSTT1*  *NQO1*  *GGT1* | aggaggcagccctggtggac  ctggttctgggacagcagggtct  tggatgagtacctggcatggca  cctcgagcaactgcagggtcac  gagcccagccaatcagcgc  ggtccgactccaccacctccc  caagcagtgctcgaagattggga  gggcgttgatgacctcagcttttc |  |
| *SERPINA1* | ggatttggtcaaggagcttgacagag  aaacg cttcatcataggcaccttcac |  |
| *TGFB1* | catgaaccggcctttcctgct  tccagccgaggtccttgcg |  |
| *FOS* | tttgcctaaccgccacgatga  aagttggcactggagacggcc |  |
| *NFKB1* | gcactgtaactgctggacccaagg  aataggcaaggtcagggtgcacc |  |
| *WISP2* | acagctgccggaacataaagactcac  caggtacatggtgtcgggcacag |  |
| *CEBPB* | aagaccgtggacaagcacagcg  gacagctgctccaccttcttctgc |  |
| *IGF1* | cacaccatgtcctcctcgcatctc  cgccctccgactgctgga |  |
| *IGF2* | ttcttggccttcgcctcgtg  gccaggtcacagctgcgga |  |
| *KISS1R* | gctgggcgacttcatgtgca  tgaggctgacagccagcgc |  |

**Supplementary Table 2.** Proteins identified by proteomics allocated to two main functional networks according to the Ingenuity Pathway Analysis (IPA). Molecules shown in bold were those identified in Table 5 (proteomics).

| ID | Molecules in Network | Score | Focus Molecules | Top Functions |
| --- | --- | --- | --- | --- |
| 1 | 26s Proteasome, Anti-inflammatory Cytokine, Cdc2, **COL1A1**, CREB-NFkB, Cytokeratin, ERK, ERK1/2, estrogen receptor, **FGA**, FOXN2, **HBB**, IL1, Jnk, KIF15, KLF3, **KRT8**, **KRT18**, KRT20, KRT72, MMP26, NFkB (complex), P38 MAPK, **PPIB**, P**RDX1**, SCUBE3, **SERPINA1**, **SERPINB2**, SERPINF2, SPRED2, TCHP, trehalose, TROAP, Vegf, **VIM** | 26 | 10 | Cell Morphology, Cellular Assembly and Organization, Cellular Compromise |
| 2 | ACTR6, ANXA6, ATP5C1, ATP5D, **ATP5F1**, ATP5H, ATP5I, ATP5J2, ATP5J, ATP5L, **CA1**, CLEC7A, CLIC1, DDR1, **EFHD1**, EHD2, FBXO11, HNRNPD, HSD17B7, IGHE, KRT33B, MAPK6, MCAM, OSMR, PDLIM1, PGRMC1, PKN1, **PNP**, PODXL, RNF41, SPRED2, **TAGLN2**, UBC, USP1, ZFP36L1 | 11 | 5 | DNA Replication, Recombination, and Repair, Energy Production, Nucleic Acid Metabolism |

**Supplementary Table 3.** Toxicological and biological functional pathways in the term human placental proteome affected by maternal smoking. Only significantly expressed functions with ≥3 proteins from the proteomic data are shown.

| **Toxicological functions** | **p-Value** | **Molecules** | **# Molecules** |
| --- | --- | --- | --- |
| Liver Cirrhosis | 4.30E-04 | COL1A1, KRT18, KRT8 | 3 |
| Hepatocellular Carcinoma | 2.78E-03 | FGA, PRDX1, SERPINA1, VIM | 4 |
| Liver Hyperplasia or Hyperproliferation | 2.78E-03 | FGA, PRDX1, SERPINA1, VIM | 4 |
| **Biological functions** | **p-Value** | **Molecules** | **# Molecules** |
| Cell Death and Survival | 1.50E-05 | COL1A1, FGA, HBB, KRT18, KRT8, PNP, PPIB, PRDX1, SERPINA1, SERPINB2, TAGLN2, VIM | 12 |
| Cellular Assembly and Organization | 5.32E-05 | KRT18, KRT8, SERPINA1 | 3 |
| Cellular Compromise | 5.32E-05 | KRT18, KRT8, SERPINA1 | 3 |
| Cell Death and Survival | 5.36E-05 | HBB, KRT18, PNP, PRDX1 | 4 |
| Cellular Movement | 6.12E-05 | COL1A1, FGA, KRT8, SERPINA1, TAGLN2, VIM | 6 |
| Hepatic System Development and Function | 7.80E-05 | COL1A1, FGA, KRT18, KRT8 | 4 |
| Digestive System Development and Function | 7.80E-05 | COL1A1, FGA, KRT18, KRT8 | 4 |
| Organ Morphology | 7.80E-05 | COL1A1, FGA, KRT18, KRT8 | 4 |
| Connective Tissue Disorders | 7.89E-05 | CA1, COL1A1, PPIB | 3 |
| Skeletal and Muscular Disorders | 7.89E-05 | CA1, COL1A1, PPIB | 3 |
| Cell Death and Survival | 8.09E-05 | COL1A1, FGA, KRT18, KRT8, PNP, PRDX1, SERPINA1, SERPINB2, TAGLN2, VIM | 10 |
| Cellular Movement | 8.16E-05 | COL1A1, FGA, KRT8, PPIB, PRDX1, SERPINA1, SERPINB2, TAGLN2, VIM | 9 |
| Organ Morphology | 2.01E-04 | HBB, PNP, PRDX1 | 3 |
| Cell Death and Survival | 2.37E-04 | HBB, PNP, PRDX1 | 3 |
| Cellular Growth and Proliferation | 2.63E-04 | ATP5F1, COL1A1, FGA, KRT8, PNP, PPIB, PRDX1, SERPINA1, SERPINB2, TAGLN2, VIM | 11 |
| Cellular Movement | 2.82E-04 | COL1A1, FGA, KRT8, PPIB, PRDX1, SERPINA1, SERPINB2, VIM | 8 |


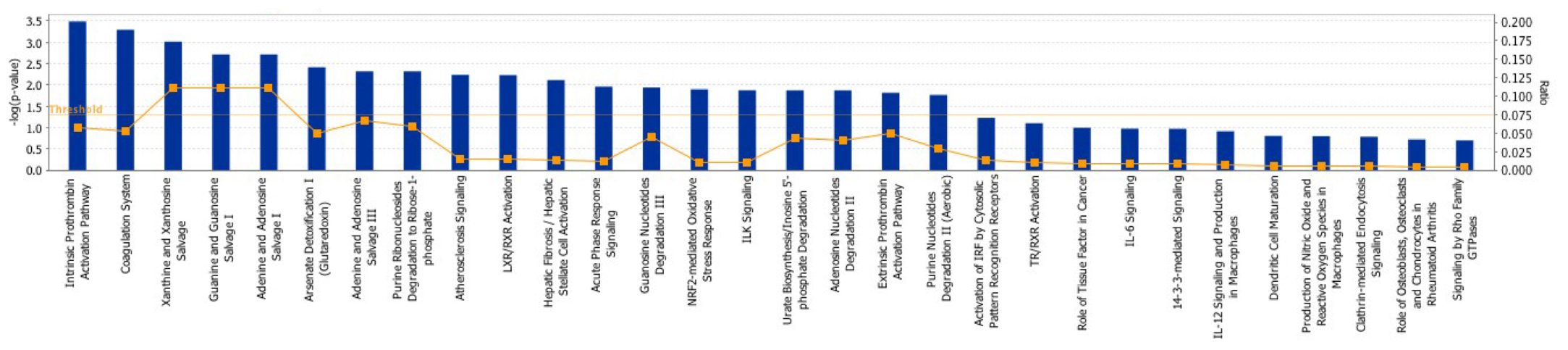


**Supplementary Figure 2.** Canonical pathways in the term human placental proteome affected by maternal smoking.
